# Enhancing Supplemental Effects of Acute Natural Antioxidant Derived from Yeast Fermentation and Vitamin C on Sports Performance in Triathlon Athletes: A Randomized, Double-Blinded, Placebo-Controlled, Crossover Trial

**DOI:** 10.3390/nu15153324

**Published:** 2023-07-26

**Authors:** Eunjoo Lee, Hun-Young Park, Sung-Woo Kim, Yerin Sun, Jae-Ho Choi, Jisoo Seo, Yanghoon Peter Jung, Ah-Jin Kim, Jisu Kim, Kiwon Lim

**Affiliations:** 1 Department of Sports Medicine and Science, Graduate School, Konkuk University, Seoul 05029, Republic of Korea; eunjooo@konkuk.ac.kr (E.L.); parkhy1980@konkuk.ac.kr (H.-Y.P.); kswrha@konkuk.ac.kr (S.-W.K.); edre82@konkuk.ac.kr (Y.S.); zas1135@konkuk.ac.kr (J.-H.C.); zxsd23@konkuk.ac.kr (J.S.); kimpro@konkuk.ac.kr (J.K.); 2 Physical Activity and Performance Institute, Konkuk University, Seoul 05029, Republic of Korea; 3 CJ CheilJedang Food & Nutrition Tech, Jung-gu, Seoul 04527, Republic of Korea; yp.jung@cj.net (Y.P.J.); ahjin.kim@cj.net (A.-J.K.); 4 Department of Physical Education, Konkuk University, Seoul 05029, Republic of Korea

**Keywords:** metabolic function, skeletal muscle oxygenation, cardiac function, antioxidant, glutathione, vitamin C, ActiveNrich, prolonged submaximal exercise, triathlon athletes, dietary supplementation, sports performance, fatigue, yeast extract, oxidative stress, synergistic effect

## Abstract

This study investigated the acute effects of natural antioxidants, derived from yeast fermentation containing glutathione and dietary vitamin C supplementation, on metabolic function, skeletal muscle oxygenation, cardiac function, and antioxidant function during submaximal exercise in middle-aged triathlon athletes. Twelve participants (aged 49.42 ± 5.9 years) completed 90 min submaximal cycling trials corresponding to 70% maximal oxygen uptake with either vitamin C and glutathione (VitC+Glu), vitamin C (VitC), glutathione (Glu) supplementation, or placebo. Metabolic function (minute ventilation, oxygen uptake, carbon dioxide output [VCO_2_], respiratory exchange ratio [RER], oxygen pulse [O_2_pulse], carbohydrate oxidation, fat oxidation, and energy expenditure), skeletal muscle oxygenation (oxidized hemoglobin and myoglobin in skeletal muscle tissue, total hemoglobin and myoglobin in skeletal muscle tissue [tHb]), cardiac function (heart rate [HR], stroke volume [SV], cardiac output, end-diastolic volume, end-systolic volume, and ejection fraction), and antioxidant function parameters (blood lactate, superoxide dismutase, catalase, glutathione peroxidases, glutathione [GSH], diacron reactive oxygen metabolite [dROM], and biological antioxidant potential [BAP]) were measured during submaximal exercise and recovery. VCO_2_, RER, HR, blood lactate after exercise, and dROM were significantly lower, and O_2_pulse, tHb, and BAP were significantly higher for VitC+Glu than for the other trials (*p* < 0.05). In conclusion, combined vitamin C and glutathione supplementation was more effective in improving metabolic function, skeletal oxygenation, cardiac function, and antioxidant function during prolonged submaximal exercise in middle-aged triathletes.

## 1. Introduction

Oxidative stress occurs when the production of reactive oxygen species (ROS) exceeds the ability of the body’s defense system to eliminate them. During exercise, the body increases its oxygen consumption to generate energy for muscle contraction. This increase in oxygen consumption can lead to the generation of ROS, such as superoxide, hydrogen peroxide, and hydroxyl radicals, as byproducts of cellular respiration [1,2,3]. Excessive ROS production during high-intensity exercise can overwhelm the antioxidant defense system of the body, leading to oxidative stress that can cause cellular damage and impair exercise performance. After short- or long-term exercise training, prominent increases in oxidative stress biomarkers, reductions in glutathione and antioxidant vitamins, accumulation of free radicals within muscles, and lipid peroxidation have been observed [4]. Moreover, elevated exercise-induced oxidative stress stimulates the expression of proinflammatory cytokines, leading to inflammation, muscle damage, and accelerated fatigue, ultimately decreasing exercise performance [5,6].

Researchers have discovered that increasing the levels of antioxidants (including glutathione, *N*-acetylcysteine, alpha-lipoic acid, and vitamins A, C, and E) in the bloodstream can help reduce oxidative stress by preventing the accumulation of free radicals in cells [7,8,9]. Several studies have investigated the potential of antioxidant supplementation (by reducing exercise-induced oxidative stress) to enhance exercise performance. Supplementation with coenzyme Q10 positively affects exercise capacity and recovery [10,11,12,13]. Several studies have suggested that polyphenols, such as quercetin [14,15] and the polyphenolic compounds found in grape extract [16], demonstrate performance-enhancing effects. 

The efficacy of vitamin C, a potent free radical scavenger, in exercise-induced oxidative stress has been studied; however, the results remain controversial. Several studies have shown that vitamin C supplementation is beneficial in regulating redox balance and reducing the production of oxidative stress biomarkers during exercise, such as lipid peroxidation and protein carbonyl [17,18,19,20]. In studies involving male athletes, the group supplemented with an antioxidant complex, including vitamin C, showed more positive exercise training effects than the placebo group [21,22], and Jourkesh et al. reported an increase in aerobic power with the combined intervention of vitamins C and E [23]. However, other studies have reported that vitamin C and/or E supplementation does not enhance exercise performance [24,25,26,27,28]. These diverse results may be due to differences in exercise protocols, participant populations, dosages, forms of supplements, duration and timing of supplementation, and methodologies used to assess oxidative stress [29]. Thus, an appropriate protocol for reducing oxidative stress and improving exercise performance has not yet been established and further research on this topic is needed.

Glutathione is a powerful antioxidant found in all cells of the human body [30]. It is synthesized from glutamate, cysteine, and glycine in the cytosol and mainly exists in its reduced form (GSH) [31]. GSH is utilized during redox reactions to eliminate ROS and is transformed into its oxidized form (GSSG). There is growing interest in interventions that can increase GSH levels, as higher concentrations of GSH have been shown to protect against cellular damage, tissue degeneration, and disease progression in various models [32]. Prolonged exercise leads to a gradual decrease in plasma and tissue glutathione levels [33,34]. This finding highlights the potential role of glutathione in maintaining aerobic metabolism and muscle contraction. Some animal studies have reported that oral glutathione supplementation positively affects exercise performance [35,36]. Aoi et al. found that when healthy men who had been supplemented with glutathione underwent 60 min of cycling exercise, they showed a lower decrease in plasma glutathione and an inhibited increase in blood lactate concentrations compared with the placebo group. This suggests that glutathione supplementation effectively improves muscle aerobic metabolism and reduces muscle fatigue during exercise [37]. Despite these positive effects, research about glutathione and exercise performance has not been sufficiently studied.

The biochemical processes of vitamin C and GSH are closely connected [38]. GSH plays a role in reducing oxidized dehydroascorbate back to ascorbate [39]. A deficiency in GSH decreases the level of ascorbate in tissues [40], and in young adults with low vitamin C intake, GSH depletion and oxidative stress have been observed [41]. Johnston et al. reported that vitamin C intake can maintain the concentration of GSH in the blood and increase antioxidant protection [42] and that vitamin C supplementation can help increase lymphocyte glutathione levels [43]. Glutathione and vitamin C may enhance each other to achieve optimal function as antioxidants and reduce oxidative stress. However, despite the biochemically intertwined relationship between vitamin C and glutathione, research on the combined intake of glutathione and vitamin C on exercise remains limited. Sastre et al. [44] reported that the combined intake of glutathione and vitamin C may prevent exercise-induced oxidative stress. Their study holds significant importance as the sole study investigating the acute combined intake of glutathione and vitamin C on exercise. However, it had a limited sample size, comprising only five participants. They provided data only on GSH in blood, GSSG in blood, and GSH:GSSG ratio without statistical analysis. As there were no additional experimental findings, it is challenging to precisely validate the effects of acute vitamin C and glutathione supplementation on exercise. Therefore, we designed our study to expand the sample size and analyze various parameters (i.e., metabolic function, skeletal muscle oxygenation, cardiac function, and antioxidant function) to investigate the effects of acute combined supplementation of vitamin C and glutathione during prolonged exercise. 

This study aimed to investigate the acute effects of natural antioxidants derived from yeast fermentation containing glutathione and dietary vitamin C on metabolic function, skeletal muscle oxygenation, cardiac function, and antioxidant function during prolonged submaximal exercise. We hypothesized that acute dietary vitamin C and glutathione supplementation may result in greater improvements in metabolic function, skeletal muscle oxygenation, cardiac function, and antioxidant function during prolonged submaximal exercise in middle-aged triathlon athletes.

## 2. Materials and Methods

### 2.1. Participants

Twelve middle-aged triathlon athletes (nine male and three female) with no history of musculoskeletal, cardiovascular, or pulmonary diseases (age, 49.42 ± 5.9 years; height, 169.6 ± 5.0 cm; fat-free mass, 52.9 ± 8.6 kg; fat mass, 10.5 ± 4.5 kg; percent body fat, 16.4 ± 5.5%; maximal oxygen uptake [VO_2max_], 45.97 ± 7.41) were recruited. The sample size was determined based on the concentration of GSH in the blood, which was a key variable in this study. Using the mean and standard deviation of results from a previous study by Silva et al. [45], the effect size was calculated using the method proposed by Cohen [46], which yielded a value of 1.111. Based on this, with α = 0.05 and a power of 0.8 (1−β), and assuming four groups with three repeated measurements each, the required sample size that satisfied these conditions was calculated to be a total of eight participants using G-power. However, to account for potential dropouts, we aimed to obtain a sample size of 12 participants. During the study, the participants were asked not to engage in any extra activities and to follow their regular diets, taking no additional vitamins or mineral supplements except those provided by the investigators. The participants were informed of the purpose and process of this study and provided consent before the start of the study. This study was approved by the Institutional Review Board of Konkuk University (7001355-202302-HR-624), Republic of Korea, and was conducted in accordance with the Declaration of Helsinki. The trial information is registered with the Clinical Research Information Service in Korea (KCT0008587). Written informed consent was obtained from all the participants.

### 2.2. Study Design

**Figure 1:**
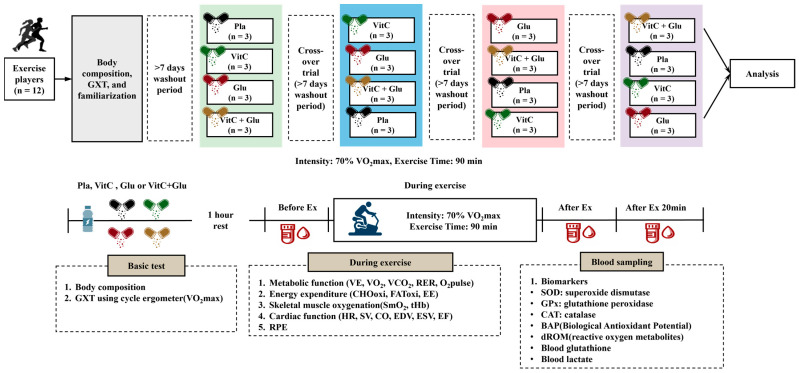
Study design. Pla: placebo; VitC: vitamin C supplement; Glu: glutathione supplement; VitC+Glu: combined vitamin C and glutathione supplement, GXT: graded exercise test; VO_2max_: maximal oxygen uptake; VE: minute ventilation; VO_2_: oxygen uptake; VCO_2_: carbon dioxide output; RER: respiratory exchange ratio; O_2_pulse: oxygen pulse; CHOoxi: carbohydrate oxidation; FAToxi: fat oxidation; EE: energy expenditure; SmO_2_: oxidized hemoglobin and myoglobin in skeletal muscle tissue; tHb: total hemoglobin and myoglobin in skeletal muscle tissue; HR: heart rate; SV: stroke volume; CO: cardiac output; EDV: end-diastolic volume; ESV: end-systolic volume; EF: ejection fraction; RPE: ratings of perceived exertion; SOD: superoxide dismutase; GPx: glutathione peroxidase; CAT: catalase; BAP: biological antioxidant potential; dROM: diacron reactive oxygen metabolite.

The design of this study, which was a double-blind, cross-over, placebo-controlled trial, is illustrated in Figure 1. All the participants visited our laboratory five times during the experimental period. The order of supplementation was determined using computer-generated randomization. Based on previous studies on acute oral supplementation, the washout period of at least 7 days between all trials was implemented to prevent carryover effects [47,48,49,50,51,52].

At the first visit, the participants arrived after an 8-hour fast to undergo body composition measurements. After stabilization, they performed a graded exercise test (GXT) using a cycle ergometer (Aerobike 75XLIII, Konami Corporation, Tokyo, Japan) to evaluate VO_2max_. The GXT protocol was started at 600 kg∙m∙min^−1^ (100 watts) for males and at 300 kg∙m∙min^−1^ (50 watts) for females, and the exercise load was increased by 150 kg∙m∙min^−1^ (25 watts) every 2 min; the pedaling speed was set to 60 rpm. The exercise intensity was set at a workload of 70% of VO_2max_ (141.7 ± 26.8 watts) on a bicycle ergometer for submaximal exercise trials.

During the remaining four visits, participants performed each exercise trial with four different supplements: placebo (Pla), vitamin C (VitC), glutathione (Glu), and combined vitamin C and glutathione (VitC+Glu) supplements. For the exercise trials, the participants arrived at the laboratory after fasting for 4 h. Upon arrival, the participants consumed a 100 mL solution containing either supplements or placebo and then rested for 1 h. They performed a 90 min bout of prolonged submaximal exercise using a cycle ergometer (Aerobike 75XLIII, Konami Corporation, Tokyo, Japan) at a predetermined intensity (workload of 70% VO_2max_) with a pedaling speed of 60 rpm. For data acquisition, blood samples were collected before exercise, immediately after exercise, and at the 20 min post-exercise time point. The following parameters were analyzed: blood lactate, superoxide dismutase (SOD), catalase (CAT), glutathione peroxidase (GPx), GSH, diacron reactive oxygen metabolite (dROM), and biological antioxidant potential (BAP). In addition, the following measurements were taken every minute during the 90 min bout of prolonged submaximal exercise: metabolic function parameters (minute ventilation [VE], oxygen uptake [VO_2_], carbon dioxide output [VCO_2_], respiratory exchange ratio [RER], oxygen pulse [O_2_pulse], carbohydrate oxidation [CHOoxi], fat oxidation [FAToxi], and energy expenditure [EE]) and cardiac function parameters (heart rate [HR], stroke volume [SV], cardiac output [CO], end-diastolic volume [EDV], end-systolic volume [ESV], and ejection fraction [EF]). Skeletal muscle oxygenation profiles included oxidized hemoglobin and myoglobin in skeletal muscle tissue (SmO_2_) and total hemoglobin and myoglobin in skeletal muscle tissue (tHb). Ratings of perceived exertion (RPE) were measured using the Borg 6–20 scale every 10 min during the 90 min bout of prolonged exercise.

All exercise trial sessions were performed in a 9 m × 7 m × 3 m (width × length × height) environmental control chamber (NCTC-1, Nara Control, Seoul, Republic of Korea) at a temperature of 23 ± 1 °C and humidity of 50 ± 5% regulated by an environmental control chamber.

### 2.3. Dietary Supplementation

The participants consumed a solution containing Pla, VitC, Glu, or VitC+Glu dissolved in 100 mL of water on 2nd, 3rd, 4th, and 5th visits. The order of dietary supplementation was randomly assigned to one of the four sequences: A, B, C, or D, as shown in Table 1. VitC contained 110 mg of vitamin C, Glu contained 252 mg of food-grade glutathione extracted from yeast (ActiveNrich^TM^, CJ CheilJedang, Seoul, Republic of Korea), and VitC+Glu contained 110 mg of vitamin C and 252 mg of food-grade glutathione extracted from yeast. The placebo was prepared to have the same taste as the three supplements. All supplements were prepared by CJ CheilJedang Food and Nutrition Tech (Seoul, Republic of Korea).

### 2.4. Measurements

#### 2.4.1. Anthropometry and Body Composition

Height was measured using a stadiometer (YM-1, KDS, Seoul, Republic of Korea), and body composition (i.e., weight, fat-free mass, fat mass, and percentage of body fat) was measured after fasting for more than 8 h using a bioelectrical impedance analysis device (Inbody 770; Inbody, Seoul, Republic of Korea).

#### 2.4.2. Metabolic Function

To analyze metabolic function, VE, VO_2_, VCO_2_, RER, and O_2_pulse were measured using a K5 auto metabolism analyzer (COSMED, Monte Savello, Italy) and a breathing valve in the form of a facemask every minute during a 90 min bout of prolonged submaximal exercise. In addition, CHOoxi, FAToxi, and EE were calculated using stoichiometric Equation (1) proposed by Jeukendrup and Wallis [53].

(1) $$CHOoxi=4.210\times {VCO}_{2}-2.962\times {VO}_{2}\;FAToxi=1.695\times {VO}_{2}-1.701\times {VCO}_{2}\;EE=4.07\times CHO+9.75\times FAToxi$$

#### 2.4.3. Skeletal Muscle Oxygenation

Regarding skeletal muscle oxygenation profiles, SmO_2_ and tHb were measured using a near-infrared spectroscopy (NIRS) system for muscle tissue (Moxy Monitor, Fortiori Design LLC, Hutchinson, MN, USA). After attaching the NIRS probe to the right vastus lateralis muscle 10–15 cm above the knee, data were recorded every 10 s from rest to the end of the 90 min bout of prolonged submaximal exercise, and an average value of 1 min was used for analysis.

#### 2.4.4. Cardiac Function

Cardiac function parameters including HR, SV, CO, EDV, ESV, and EF were assessed noninvasively using a thoracic bioelectrical impedance device (PhysioFlow PF-05, Manatec Biomedical, Paris, France) during a 90 min bout of prolonged submaximal exercise. Data were recorded every 10 s from rest to the end of the exercise, and an average value of 1 min was used for analysis.

##### 2.4.5. Blood Samples

Blood samples were obtained before exercise, immediately after exercise, and 20 min after the end of exercise. An 8 mL sample of venous blood was collected in a serum separate tube. The samples were then flipped approximately five times and left to stand upright for approximately 30 min. Subsequently, they were centrifuged at 3000× *g* for 15 min to separate the serum. The serum was stored at −20 °C until the time of the assay. The levels of SOD, CAT, GPx, GSH, BAP, and dROM were analyzed by the Seegene Medical Foundation (Seoul, Republic of Korea) upon request. Serum GSH level and SOD, CAT, and GPx activity were assessed using a sandwich enzyme-linked immunosorbent assay (Human Glutathione (GSH) ELISA Kit, AFG Bioscience, Northbrook, IL, USA; Human total SOD ELISA Kit, Wuhan Fine Biotech, Wuhan, China; Human Catalase (CAT) ELISA Kit, Cusabio, Houston, TX, USA; and Human total GPX1 ELISA Kit, Wuhan Fine Biotech, Wuhan, China). The prooxidant status, dROM, antioxidant status, and BAP were quantified photometrically using Diacron’s analysis kit (dROMs and BAP test kit, Diacron International, Grosseto, Italy) on a Roche Hitachi 912 Chemistry Analyzer (Roche Diagnostics, Indianapolis, IN, USA). Blood lactate levels were measured using a Lactate Pro 2 (Arkray, Kyoto, Japan).

##### 2.4.6. RPE

The participants were shown the Borg scale every 10 min during a 90 min bout of prolonged submaximal exercise and were asked to indicate their perceived exertion level by pointing to a corresponding number on the scale using their fingers.

### 2.5. Statistical Analysis

All statistical analyses were performed using SPSS version 25.0 (IBM Corp., Armonk, NY, USA) for Windows. Data are reported as the mean ± standard deviation. The normality of the distribution of all acquired data was verified using the Shapiro–Wilk W-test prior to the parametric tests. To verify the difference in efficacy between each supplement, which was the main purpose of the present study, metabolic function, skeletal muscle oxygenation, and cardiac function were analyzed using one-way analysis of variance (ANOVA) with repeated measures. Two-way ANOVA with repeated measures was used to assess the presence of interactions (supplement × time) and the main effects (supplement or time) on RPE, blood lactate, and antioxidant biomarkers. When ANOVA revealed a significant interaction or main effect within the trial (supplement), a Bonferroni post hoc test was used to identify within-trial differences at each time point. The level of significance was set a priori at *p* < 0.05.

## 3. Results

### 3.1. Metabolic Function

**Figure 2:**
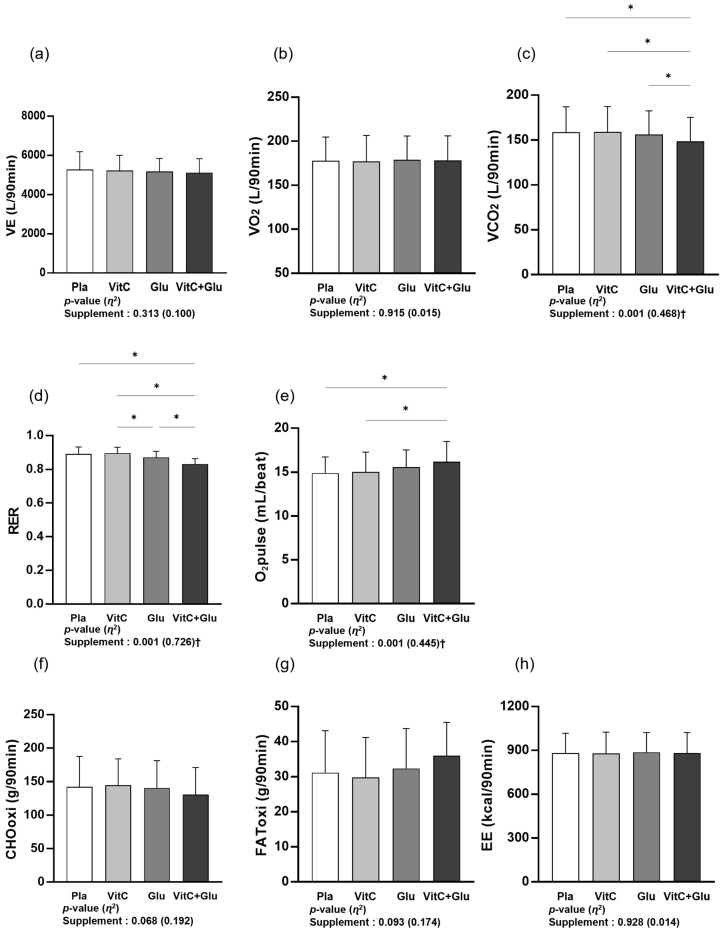
The differences in metabolic function parameters between the four trials (supplements) during a 90 min bout of prolonged submaximal exercise. (**a**) minute ventilation (VE), (**b**) oxygen uptake (VO_2_), (**c**) carbon dioxide output (VCO_2_), (**d**) respiratory ex-change ratio (RER), (**e**) oxygen pulse (O_2_) pulse, (**f**) carbohydrate oxidation (CHOoxi), (**g**) fat oxidation (FAToxi), and (**h**) energy expenditure (EE). Pla: placebo; VitC: vitamin C supplement; Glu: glutathione supplement; VitC+Glu: combined vitamin C and glutathione supplement. The bars indicate the mean ± standard deviation. * indicates significant difference from supplement (*p* < 0.05). † indicates significant main effect within trial (*p* < 0.05).

Figure 2 depicts the differences in metabolic function parameters between the four trials (supplements) during a 90 min bout of prolonged submaximal exercise. No significant main effect within trial was observed for VE, VO_2_, CHOoxi, FAToxi, or EE; however, VCO_2_ (*p* < 0.001, *η*^2^ = 0.468), RER (*p* < 0.001, *η*^2^ = 0.726), and O_2_pulse (*p* < 0.001, *η*^2^ = 0.445) showed significant main effects within trial. In the post hoc analysis, VCO_2_ was significantly lower for VitC+Glu than for the other trials. The RER was also significantly lower for VitC+Glu than for the other trials, and Glu showed a significantly lower VCO_2_ than VitC. O_2_pulse was significantly higher for VitC+Glu than for placebo and VitC alone. In addition, CHOoxi showed a lower trend (*p* = 0.068, *η*^2^ = 0.192) and FAToxi showed a higher trend (*p* = 0.093, *η*^2^ = 0.174) in VitC+Glu than in the other trials.

### 3.2. Skeletal Muscle Oxygenation

**Figure 3:**
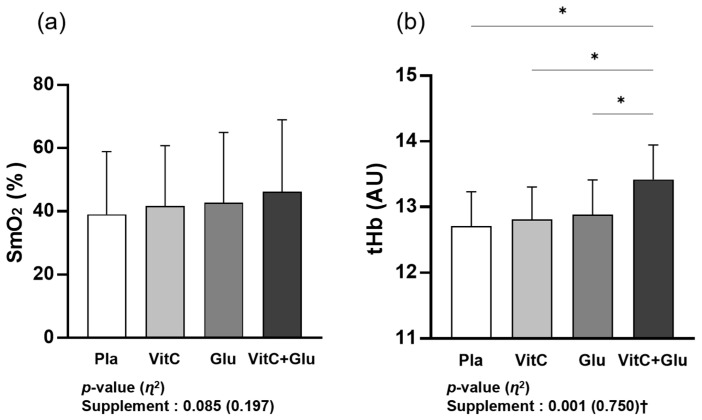
The differences in skeletal muscle oxygenation parameters between the four trials (supplements) during a 90 min bout of prolonged submaximal exercise: (**a**) hemoglobin and myoglobin in skeletal muscle tissue; SmO_2_ and (**b**) total hemoglobin and myoglobin in skeletal muscle tissue; tHb. Pla: placebo; VitC: vitamin C supplement; Glu: glutathione supplement; VitC+Glu: combined vitamin C and glutathione supplement. The bars indicate the mean ± standard deviation. * indicates significant difference from supplement (*p* < 0.05). † indicates significant main effect within trial (*p* < 0.05).

As shown in Figure 3, there was no significant main effect within trial (supplement) for SmO_2_; however, it showed a higher trend in VitC+Glu than in the other trials (*p* = 0.085, *η*^2^ = 0.197). Additionally, tHb showed a significant main effect within trial (*p* < 0.001, *η*^2^ = 0.750). In the post hoc analysis, tHb was significantly higher for VitC+Glu than for the other trials.

### 3.3. Cardiac Function

**Figure 4:**
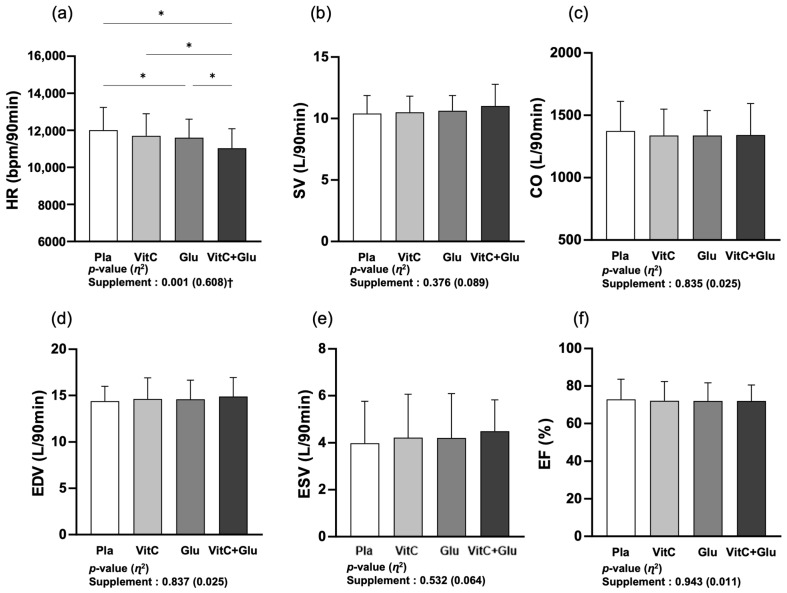
The differences in cardiac function parameters between the four trials (supplements) during a 90 min bout of prolonged submaximal exercise. (**a**) heart rate (HR), (**b**) stroke volume (SV), (**c**) cardiac output (CO), (**d**) end-diastolic volume (EDV), (**e**) end-systolic volume (ESV), and (**f**) ejection fraction (EF). Pla: placebo; VitC: vitamin C supplement; Glu: glutathione supplement; VitC+Glu: combined vitamin C and glutathione supplement. The bars indicate the mean ± standard deviation. * indicates significant difference from supplement (*p* < 0.05). † indicates significant main effect within trial (*p* < 0.05).

Figure 4 presents the differences in cardiac function parameters between the four trials (supplements) during a 90 min bout of prolonged submaximal exercise. No significant main effect within trial was observed for SV, CO, EDV, ESV, or EF; however, HR showed a significant main effect within the trial (*p* < 0.001, *η*^2^ = 0.608). In the post hoc analysis, HR was significantly lower for VitC+Glu than for the other trials, and Glu showed as significantly lower than placebo.

### 3.4. Blood Samples

**Figure 5:**
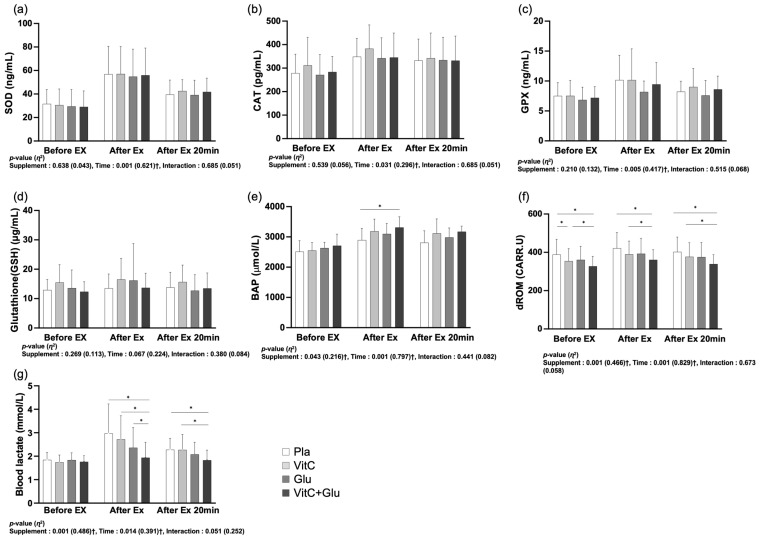
The differences in antioxidant biomarker parameters between the four trials (supplements) before, after, and 20 min after a 90 min bout of prolonged submaximal exercise. (**a**) superoxide dismutase (SOD), (**b**) catalase (CAT), (**c**) glutathione peroxidase (GPx), (**d**) glutathione (GSH), (**e**) biological antioxidant potential (BAP), (**f**) diacron reactive oxygen metabolite (dROM), and (**g**) blood lactate. Pla: placebo; VitC: vitamin C supplement; Glu: glutathione supplement; VitC+Glu: combined vitamin C and glutathione supplement. The bars indicate the mean ± standard deviation. * indicates significant difference (*p* < 0.05). † indicates significant main effect (*p* < 0.05).

As depicted in Figure 5, there was no significant interaction between time and trial (supplement) and the main effect within trial (supplement) for SOD, CAT, GPX, and glutathione. However, blood lactate (*p* < 0.001, *η*^2^ = 0.486), BAP (*p* = 0.043, *η*^2^ = 0.216), and dROM (*p* = 0.001, *η*^2^ = 0.466) showed significant main effects within trial. In the post hoc analysis, blood lactate was significantly lower for VitC+Glu than for the other trials at the time point after exercise, and it was also significantly lower for VitC+Glu than for placebo and VitC at 20 min after the end of exercise. BAP was significantly higher for VitC+Glu than for placebo at the time point after exercise. The dROM was significantly lower for the VitC+Glu group than for the placebo and VitC groups at the time point before exercise, after exercise and 20 min after the end of exercise. VitC also showed a significantly lower dROM than placebo at the time point before exercise.

### 3.5. RPE

**Figure 6:**
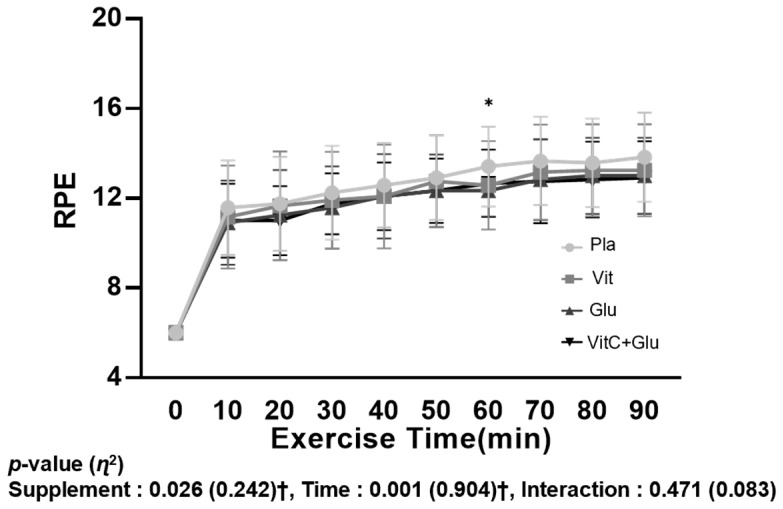
Ratings of perceived exertion (RPE) during a 90 min bout of prolonged submaximal exercise. Pla: placebo; VitC: vitamin C supplement; Glu: glutathione supplement; VitC+Glu: combined vitamin C and glutathione supplement. The bars indicate the mean ± standard deviation. * indicates significant difference between Pla and Glu *(p* < 0.05). † indicates significant main effect (*p* < 0.05).

The RPE indicated no significant interaction between time and trial (supplement); however, there was a significant main effect within trial (*p* = 0.026, *η*^2^ = 0.242). Post hoc analysis confirmed that there was a significant difference between Pla and Glu at 60 min during a 90 min bout of prolonged submaximal exercise; however, it seems difficult to assign physiological and biological meaning (Figure 6).

## 4. Discussion

Although the individual effects of glutathione and vitamin C are well known, research on their combined intake is scarce, despite their interdependent relationship in the body. In a study conducted by Sastre et al. [44], after 7 days of oral supplementation with 1 g glutathione and 2 g vitamin C, a positive change in the GSSG/GSH ratio was observed after maximal exercise. Furthermore, the same study indicated a positive linear relationship between the GSSG/GSH and lactate/pyruvate ratios before and after exercise and during the recovery phase, suggesting that the combined intake of glutathione and vitamin C could prevent exercise-induced muscle damage. However, this study had a small sample size of only five participants, and no statistical analysis was performed. Additionally, the study did not analyze parameters other than the GSSG/GSH ratio, making it difficult to properly evaluate the effects of the combined intake of glutathione and vitamin C. This [44] was the only study we found that examined the effects of glutathione and vitamin C supplementation on exercise. Therefore, we endeavored to investigate the impact of the combined intake of glutathione and vitamin C on submaximal exercise using a well-controlled study design with a larger sample size and analysis of various variables. The present study hypothesized that acute dietary vitamin C and glutathione supplementation would result in greater improvements in metabolic function, skeletal muscle oxygenation, cardiac function, and antioxidant function during prolonged submaximal exercise in middle-aged triathlon athletes. Consistent with this hypothesis, when participants performed submaximal exercise after acute VitC+Glu supplementation, a greater response in metabolic function (e.g., lower VCO_2_ and RER, and higher O_2_pulse), skeletal oxygenation (e.g., higher tHb), cardiac function (e.g., lower HR level), and antioxidant function (e.g., lower blood lactate and dROM, and higher BAP) was observed compared with trials for the other supplements or placebo.

### 4.1. Metabolic Function

Regarding metabolic function, during prolonged submaximal exercise with the same workload, there was no difference in VE and VO_2_ among supplementations, but VCO_2_ was significantly lower when taking VitC+Glu, as well as RER. Although not statistically significant, there was a trend towards higher FAToxi and lower CHOoxi in the VitC+Glu trial. These findings suggest that taking VitC+Glu before prolonged submaximal exercise may increase the reliance on fat oxidation as an energy source and conserve carbohydrate stores. These findings were consistent with those of previous studies. Johnston et al. [54] reported that adults with insufficient vitamin C levels showed 25% less fat oxidation during submaximal exercise than healthy individuals. When the same individuals were supplemented with vitamin C for 4 weeks, their serum vitamin C levels increased to normal levels, and fat oxidation during exercise increased. This indicates a strong relationship between the level of vitamin C in the bloodstream and fat oxidation during submaximal exercise. Although serum vitamin C levels were not analyzed in this study, the quantity of total antioxidants, including vitamin C, could be determined using the BAP test. BAP after 90 min of prolonged submaximal exercise was significantly higher in the VitC+Glu group than in the other groups. This suggests that an increase in the quantity of total antioxidants may lead to an increase in fat oxidation. Søndergård et al. [55] reported that insulin sensitivity in adults with obesity increased after 3 weeks of glutathione supplementation. Increased insulin sensitivity promotes fat oxidation during exercise [56]. The greater improvement observed with the combined intake of vitamin C and glutathione, compared with that observed with their individual consumption, is thought to be due to the additive effect of these two antioxidants within the body.

### 4.2. Skeletal Muscle Oxygenation

Antioxidant supplementation can remove excess ROS, reduce inflammation, and stabilize the oxidative-reductive environment, thereby improving skeletal muscle blood flow and oxygen utilization capacity [57,58]. The changes in SmO_2_ and tHb allowed us to investigate the dynamic equilibrium between the oxygen supply through the microcirculation in a specific region and its utilization by mitochondria [59]. In skeletal muscle oxygenation, a higher trend of SmO_2_ and significantly higher tHb was observed with VitC+Glu supplementation. A higher tHb may be associated with enhanced oxidative phosphorylation, that is, a reduction in phosphocreatine costs during exercise because of its high ability to deliver oxygen to muscle tissue [60,61]. This reduction in phosphocreatine re-synthesis could lead to decreased oxygen consumption (higher SmO_2_) during exercise, indicating an improvement in oxidative phosphorylation. Consequently, it is reasonable to expect an enhancement in aerobic exercise capacity [62]. While no studies were found that measured skeletal muscle oxygen saturation after the intake of glutathione or vitamin C during exercise, one study examined skeletal muscle oxygen saturation during exercise following the combined intake of other antioxidants such as anthocyanin and bromelain. In this study [63], it was observed that the intake of antioxidants improved the tissue saturation index as well as the concentrations of oxygenated hemoglobin and deoxygenated hemoglobin in the skeletal muscles, indicating an improvement in oxygen utilization in the muscles.

### 4.3. Cardiac Function

Cardiac function parameters such as SV, CO, EDV, ESV, and EF did not show any significant differences between the different supplement and placebo trials. However, HR was significantly lower in the VitC+Glu trial than in the other supplement and placebo trials. A lower HR at the same intensity is thought to indicate an improvement in exercise economy. This can be attributed to the altered myocardial cellular metabolism, which may result in more efficient energy production or utilization, leading to improved energy efficiency [64]. In a previous study, similar to our findings, a slight decrease in HR during exercise was observed after a 2-week supplementation with vitamin C; however, the difference was not statistically significant [19]. Scalzo et al. [65] reported that the infusion of vitamin C had a positive effect on cardiac function in both healthy adults and patients with type 2 diabetes. However, it did not improve maximal exercise capacity. Previous studies [66,67] have reported a significant association between ROS and cardiovascular diseases and argued that glutathione, the most abundant intracellular antioxidant, plays a crucial role in ROS scavenging.

### 4.4. Antioxidant Function and Blood Lactate

Humans possess antioxidants to protect themselves from free radicals, and they can be categorized as enzymatic and non-enzymatic antioxidants. SOD, CAT, and GPx are the most effective enzymatic antioxidants that maintain physiological homeostasis by eliminating free radicals and ROS [68]. Non-enzymatic antioxidants include vitamin E, vitamin C, vitamin B_6_, beta-carotene, selenium, *N*-acetylcysteine, and others. Unlike enzymatic antioxidants, non-enzymatic antioxidants need to be obtained from external sources, and when combined with enzymatic antioxidants, they can amplify the antioxidant effects by participating in a chain reaction [69]. Athletes commonly use non-enzymatic antioxidants such as vitamin A, E, C, and glutathione as a primary means to counteract oxidative stress during exercise [3]. In this study, we examined the effects of non-enzymatic antioxidants, specifically vitamin C and glutathione, on antioxidant biomarkers, including endogenous antioxidant enzymes, within the body. We found no significant differences in the serum levels of SOD, CAT, GPX, and GSH between the supplement and placebo trials. A previous study [44] conducted in mice showed a tendency for higher blood glutathione levels after glutathione and vitamin C supplementation during maximum exercise, although the difference was not statistically significant. In this study, the consumption of VitC+Glu tended to increase blood glutathione levels at all three time points (before, after, and 20 min after exercise); however, the difference was not significant. Khassaf et al. [70] reported improvements in SOD and CAT levels after an 8-week vitamin C supplementation, which contradicts the findings of our study. Yfanti et al. [71] reported an increased expression of antioxidant enzyme mRNA following an 8-week combination supplementation of vitamins C and E; however, they did not observe changes in antioxidant enzyme levels at the protein level. They suggested that this discrepancy could be due to technical limitations in the analysis or differences between mRNA and protein turnover rates. It cannot be concluded that antioxidant supplementation had no effect on exercise performance simply because no changes in blood components were observed. Leeuwenburgh et al. [35] reported improved aerobic exercise performance despite no positive effects on blood glutathione content or the redox system following glutathione supplementation in animal studies. This implies that gene expression or protein level analyses may not always correspond to changes in exercise performance. Additionally, Park et al. [72] stated that it is difficult to confirm an increase in blood glutathione levels using conventional glutathione testing methods and suggested the use of protein-bound glutathione detection methods. In contrast to previous studies [73,74] which found no effect of glutathione supplementation on blood glutathione levels, their study showed an increase in the concentration of protein-bound glutathione after oral administration of glutathione. However, we did not analyze it using this method in our study, and the lack of significant differences in blood glutathione levels may also be due to technical reasons.

The BAP measures the total oxidative power of the blood by assessing the concentration of antioxidants (e.g., vitamin C). Before exercise, there was no significant difference between the trials; however, immediately after exercise, there was a statistically significant increase in BAP levels in the VitC+Glu trial compared with BAP levels in the placebo trial. dROM is a measure of the concentration of acidifying substances, including hydroperoxide, and a low value indicates slower generation of ROS or faster elimination of ROS than its production. In this study, there was a significant decrease in dROM levels in the VitC+Glu trial at all three time points compared with dROM levels in the VitC or placebo trials. This indicated that the intake of VitC+Glu increased the concentration of total antioxidants in the blood, leading to a decrease in the concentration of peroxides. Thus, it can be inferred that antioxidant capacity was enhanced. Kelly et al. [75] found that plasma vitamin C concentrations were significantly higher after acute vitamin C intake than after placebo intake at 2, 4, 6, and 8 h. This level is similar to, or even higher than, the levels achieved with long-term administration of lower amounts of vitamin C, as reported by Wilkinson et al. [76]. Because the BAP measures the concentration of antioxidant substances in the blood, such as vitamin C, it can be considered to have similar results to our study. Wilkinson et al. [76] reported no changes in the biomarkers of oxidative damage after vitamin C intake, which is contradictory to our study, which showed a positive effect on the levels of dROM, an indicator of peroxide concentration. Indeed, the discrepancy in results between previous studies and our study could be attributed to the fact that previous studies focused solely on vitamin C supplementation, whereas our study involved combined treatment with glutathione and vitamin C. The significantly lower levels of dROM in the VitC+Glu trial compared with those in the VitC trial further supports this idea.

In this study, the blood lactate concentration after prolonged submaximal exercise was significantly lower when VitC+Glu was consumed prior to exercise. Lactate concentration is a measure of the balance between lactate production and elimination. A previous study [77] showed that glutathione levels in red blood cells are associated with vasodilation, and Johnston et al. [42] reported that vitamin C can increase the levels of glutathione in red blood cells. Based on these findings, a combination of vitamin C and glutathione supplementation may have a positive effect on vasodilation, potentially leading to improved microcirculation and the removal of lactic acid from the muscles. This could result in increased muscle blood flow or the upregulation of lactate transport mechanisms [78,79], which may contribute to decreased lactate accumulation. Furthermore, these findings are consistent with those of previous studies that reported a decrease in lactate levels during exercise following the consumption of vitamin C or glutathione. Goldfarb et al. [19] reported a reduction in lactate levels after a 2-week supplementation with vitamin C. Although lactate analysis was not performed directly in this study, Sastre et al. [44] demonstrated a positive correlation between the GSSG:GSH ratio and the lactate:pyruvate ratio (non-supplementation trial). Moreover, they reported a decrease in the GSSG:GSH ratio following the combination treatment with glutathione and vitamin C, implying a potential decrease in lactate accumulation.

### 4.5. RPE

In this study, except at the 60 min time point, there were no significant differences in RPE between the supplementation trials. However, Vidal et al. reported improvements in RPE during a 5 km time trial after consumption of a composite antioxidant supplement compared to placebo [80]. Our study involved a longer exercise duration of 90 min and a consistent exercise intensity at 70%VO_2max_, which might have limited the increase in RPE beyond a certain level, given the specialized nature of long-duration exercise in triathlon events. Although there were differences in heart rate based on the supplementation during the exercise at the same intensity, these differences were not perceived to be significant by the individuals.

### 4.6. Strength and Limitation

Although glutathione and vitamin C are complementary and play important roles in antioxidant mechanisms, studies on the combined intake of these two substances and exercise are limited. The clinical significance and applicability of this study lie in its findings related to the combined supplementation of vitamin C and glutathione middle-aged triathletes. These findings highlight the potential benefits of such supplementation in enhancing various aspects of exercise performance, including metabolic function, skeletal muscle oxygenation, cardiac function, and antioxidant capacity. By demonstrating the superiority of the combined supplementation over single vitamin C or glutathione supplementation, our study opens up avenues for further research and practical applications in the field of sports and exercise science. These findings could be valuable in guiding supplementation and optimizing performance strategies for middle-aged triathletes and potentially other athletes with similar exercise demands and physiological profiles. However, further investigations and validation studies are warranted to establish specific dosing recommendations and assess long-term effects for a broader range of athletes and athletic contexts.

## 5. Conclusions

Our study demonstrated that a combination of vitamin C and natural antioxidant (ActiveNrich^TM^, CJ CheilJedang, Seoul, Republic of Korea) acute supplementation was more effective for metabolic function (e.g., lower VCO_2_ and RER, and higher O_2_pulse), skeletal oxygenation (e.g., higher tHb), cardiac function (e.g., lower HR level), and antioxidant function (e.g., lower blood lactate and dROM, and higher BAP) during prolonged submaximal exercise in middle-aged triathletes than a single supplementation of vitamin C.

## Figures and Tables

**Table 1:** Randomly assigned order of dietary supplementation: A, B, C, or D.

Sequence	1st Visit	2nd Visit	3rd Visit	4th Visit	5th Visit
A (n = 3)	-	Pla	VitC	Glu	VitC+Glu
B (n = 3)	-	VitC	Glu	VitC+Glu	Pla
C (n = 3)	-	Glu	VitC+Glu	Pla	VitC
D (n = 3)	-	VitC+Glu	VitC	Glu	Glu

Note: Pla: placebo; VitC: vitamin C supplement; Glu: glutathione supplement; VitC+Glu: combined vitamin C and glutathione supplement.

## Data Availability

The data that support the findings of this study are available from the corresponding author upon reasonable request.
